# Organic molecule fluorescence as an experimental test-bed for quantum jumps in thermodynamics

**DOI:** 10.1098/rspa.2017.0099

**Published:** 2017-08-30

**Authors:** Cormac Browne, Tristan Farrow, Oscar C. O. Dahlsten, Robert A. Taylor, Vedral Vlatko

**Affiliations:** 1 Clarendon Laboratory, University of Oxford, Parks Road, Oxford OX1 3PU, UK; 2 Center for Quantum Technologies, National University of Singapore, Republic of Singapore; 3 Department of Physics, South University of Science and Technology (SUSTech), Shenzhen, People’s Republic of China; 4 London Institute, 35a South Street Mayfair, London, UK; 5 Department of Physics, National University of Singapore, 2 Science Drive 3, 117542, Singapore; 6 Center for Quantum Information, IIIS, Tsinghua University, Beijing 100084, People’s Republic of China

**Keywords:** quantum thermodynamics, quantum information, spectroscopy, dibenzoterrylene, quantum optics

## Abstract

We demonstrate with an experiment how molecules are a natural test bed for probing fundamental quantum thermodynamics. Single-molecule spectroscopy has undergone transformative change in the past decade with the advent of techniques permitting individual molecules to be distinguished and probed. We demonstrate that the quantum Jarzynski equality for heat is satisfied in this set-up by considering the time-resolved emission spectrum of organic molecules as arising from quantum jumps between states. This relates the heat dissipated into the environment to the free energy difference between the initial and final state. We demonstrate also how utilizing the quantum Jarzynski equality allows for the detection of energy shifts within a molecule, beyond the relative shift.

## 1. Introduction

Marrying the language of thermodynamics with quantum phenomena is giving rise to a quantum thermodynamics whose development defines a new frontier where the transfer of energy at the level of individual quantum objects can now be studied. The success of thermodynamics owes much to being open to experimental testing in a variety of systems [1,2]. The extension of thermodynamics has produced a substantial body of theoretical results, such as those from a resource theory perspective [3–5], single-shot thermodynamics [6–9], as well as investigations into quantum engines and refrigerators [10–13]. These have included systems operating in the quantum regime and exhibiting features like quantum coherence, such as quantum dots [14]. Here we seek to demonstrate that the fluorescence of organic molecules forms a natural test bed for these theories. Our result provides an important proof of concept for the validity of quantum thermodynamics in this regime, and opens the door for more advanced tests of the theory.

Non-equilibrium thermodynamics is evolving rapidly, and a key result is the Jarzynski equality [1,2,15,16]. This concerns the probability distribution of work into a driven system in contact with a heat bath. It essentially equates the average of the exponential work with something which is constant regardless of the driving rate. That constant is the exponential of the equilibrium free energy, i.e. the free energy between a thermal state at the initial boundary conditions and at the final. A key use of this equation is to determine the equilibrium free energy difference from non-equilibrium experiments [17–19]. The equation also holds for quantum systems [20–22]. One important area within this field, which has received a large amount of recent attention, is the development of experimental techniques and protocols which can test the theoretical predictions [23,24].

Parallel developments in physical chemistry with the advent of single-molecule spectroscopy [25,26] over the past decade have opened up unprecedented opportunities for the study of single quantum systems. Unlike studies in bulk where spectroscopic signatures are washed out by the averaging effect of ensembles, these novel techniques now allow individual molecules to be identified, tracked and probed. Thus, it is becoming possible to study energy transfers at the level of single-molecules, some of which offer ideal test-beds owing to their well-defined spectroscopic signatures and quantum state dynamics. Their properties are extremely reproducible, more so than rival quantum systems such as quantum dots and nanocrystals [25,26].

**Figure 1.**
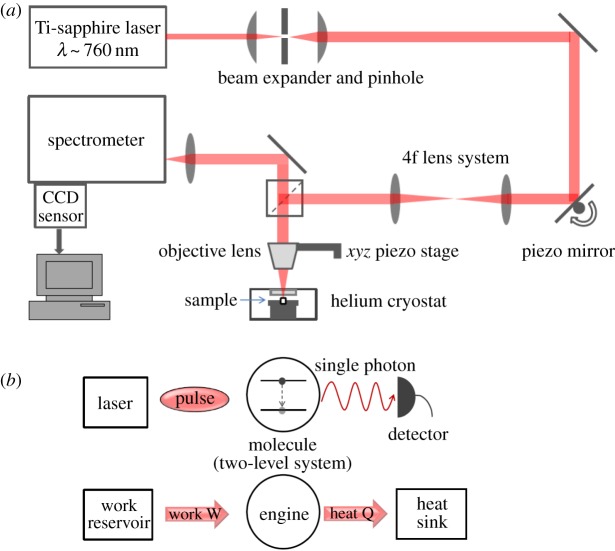
(*a*) Layout of the experimental equipment used to generate and record photoluminescence from organic molecules. (*b*) This abstraction of the experiment highlights the correspondence between a two-level molecule driven by laser pulses generating single photons from decaying electronic states with our thermodynamical representation of the process below it. (Online version in colour.)

In this work, we connect these two approaches, namely quantum thermodynamics and molecular spectroscopy, by considering the spontaneous emission of photons from a single excited organic molecule as a thermodynamic process. We determine the probability distribution of work and heat in the experiment (figure 1) and find that it satisfies the Jarzynski relation for dissipative quantum dynamics [22]. A key point is to treat the free space around the molecule as an extremely low excitation heat bath, and the fluorescent light as heat transferred into that environment. The laser applied initially corresponds to the driving force, as depicted in figure 1.

**Figure 2.**
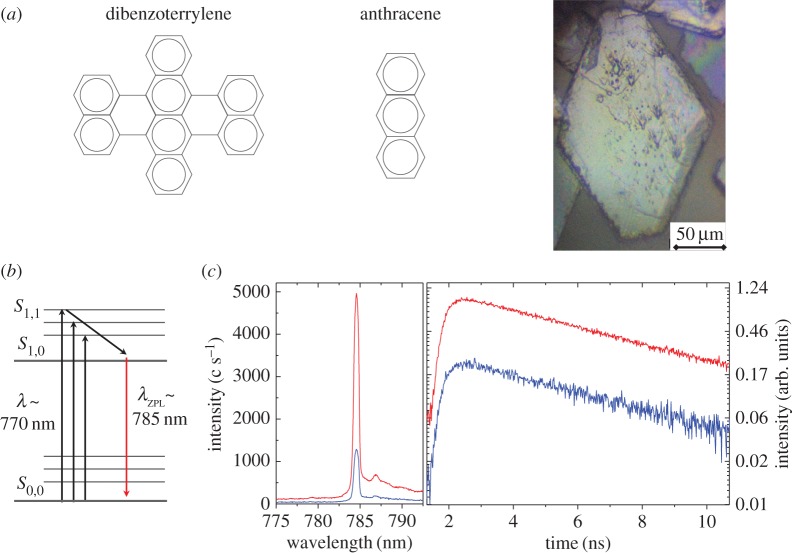
(*a*) The planar structure of dibenzoterrylene (DBT) and its host crystal anthracene exhibit a high degree of symmetry that is characteristic of organic dyes giving rise to atom-like optical spectra arising from a delocalized *π*-bond. The microscope image at the right of a typical flake of crystalline anthracene lightly doped with DBT was obtained under white light illumination. The crystal’s thickness of a few hundred nanometres maintains its transluscent appearance. (*b*) The energy-level diagram of DBT is an effective two-level system with a zero-phonon line (ZPL) transition at *λ*∼785 nm in anthracene at cryogenic temperatures. (*c*) At the left, photoluminescence from DBT generated by quasi-resonant pumping on the ZPL at a temperature of 4 K with a Ti-sapphire laser with *λ*=760 nm measured for two laser output powers, 1 mW (blue) and 4 mW (red). A Lorentzian fit of the sharp peaks at *λ*∼785 nm yields a FWHM of 27 MHz that becomes lifetime-limited at temperatures below 1.8 K. At right, waveforms of the decay of the excited electronic state at *λ*∼785 nm in DBT at temperature ∼4 K obtained by time-resolved fluorescence spectroscopy at the two excitation powers. Fitting the exponential decay curve yields a lifetime of 5 ns for the state.

Our molecule of choice is dibenzoterrylene (DBT) embedded in anthracene (figure 2), a solid crystalline host. The system offers an ideal experimental test bed that is robust, readily available and accessible to infrared spectroscopy, presenting an energy level structure with atom-like sharp two-level transitions typical of organic fluorescent dyes despite the relative complexity of the molecules. Their chemical structure consists of corrugated planar assemblies of aromatic hydrocarbons (figure 2*a*) that dopes anthracene via two insertion sites, or defects, in the extended crystal lattice, giving rise to two distinct spectral lines at *λ*∼785 nm and 795 nm at cryogenic temperatures. In this study, we probe the brighter blue-shifted site emitting at 785 nm.

Samples were prepared by collaborators [27] by co-sublimating DBT and anthracene from two differentially heated crucibles at their respective sublimation points, leading to a vapour admixture from which doped flakes crystallized on a cold finger in vacuum. We prepared control samples by crystallization from a supersaturated solution of DBT and anthracene, with nominal doping density ∼10^−4^ M, dissolved in toluene and diethylether, respectively, while heated gently above room temperature. Gradual cooling and evaporation of the admixture not more than a few millimetres deep yielded doped crystal flakes approximately 100 μm in diameter and sub-micrometre thickness (figure 2*a*). DBT molecules embedded more than a few tens of nanometres below the surface of the crystal remain photostable and robust to bleaching without needing further treatment prior to exposure to laser radiation.

Both techniques yielded comparable samples, with the co-sublimation technique proving particularly suited for controlling the density and distribution of dopant. The energetics of crystal formation from solution, coupled to the uneven local cooling rates in the solution, resulted in a doping concentration that varied arbitrarily across the crystal. Scanning the sample surface with resonant radiation showed that certain regions contained one or less molecule per square micrometre, while others had high clustering densities greater than 10^−4^ M.

We used a tunable Ti-sapphire laser to generate photoluminescence from DBT at temperature ∼4 K with the sample confined in a continuous helium gas flow cryostat to suppress spectral broadening (figure 1*a*). A spectrometer and CCD analysed the photoluminescence and a 4f lens system allowed us to move the laser spot across the sample using a piezo-actuated mirror without disturbing the position of the sample itself. An objective lens mounted on a piezoelectric stage fine-tuned the focus of the laser beam, and conversely allowed us to defocus the illumination to a larger area and to probe more molecules simultaneously using a laser signal that was blue-shifted with respect to resonant frequencies.

It is possible to discriminate the emission from individual molecules in an ensemble under illumination by using resonant excitation thanks to inhomogeneous broadening due to local strain variations across the crystal lattice. Quasi-resonant optical pumping on the ZPL transition (figure 2*b*) generates an excited state that decays non-radiatively within picoseconds into the first excited state *S*_1,0_. Decay from *S*_1,0_ into the ground state *S*_0,0_ generates a sharp lifetime-limited peak, which, for a single state in a molecule, arises from the emission of a single-photon in each decay cycle. Here we used quasi-resonant radiation with *λ*∼760 nm to reduce contributions from multiple molecules. We measured the width of the peak at *λ*∼785 nm in our sample at a temperature ∼4 K to be 27 MHz (figure 2*c*), approaching the single-molecule limit [27], and determined the lifetime of the state using time-resolved spectroscopy. The technique relies on time-correlated single photon counting of pulses generated by exciting the sample with a detuned 730 nm laser pulsed at a repetition rate of 80 MHz. The waveforms in figure 2*c*, obtained at two excitation powers, show the probability distribution of the arrival times of single photons from the decaying state rather than the shape of an optical signal. A fit of the exponential curves yields a decay time constant of 5 ns for the state corresponding to the peak at approximately 785 nm. Important to note is that the probability of detecting more than one photon per excitation period is negligible.

The experimental set-up in fact maps very naturally to the model used by Crooks in deriving the Jarzynski relation for dissipative quantum dynamics [22], and we shall use the same definitions of work and heat. In a dissipative system, the work cannot just be determined by initial and final measurements of the system’s energy. The total change in energy of the system *δU*=*Q*+*W* is equal to the work *W* applied via the time-dependent perturbation plus the flow of heat *Q* from the environment. While one may consider measuring the system’s energy throughout and thereby determine the trajectory and the heat and work from that, e.g. as in [21], Crooks’ approach avoids the associated measurement disturbance. The idea is to measure the heat flow to the environment without directly measuring the energy eigenstate of the system, except for initial and final energy measurements. The initial and final energy measurements do not impact the work distribution as (i) initially, the system is in a thermal state and thus diagonal in the energy basis, and (ii) the final measurement is after the time-dependent perturbation. The Jarzynski equality is then effectively re-expressed as

$$\langle e^{-\beta W}\rangle =\langle e^{-\beta E_{f}}\rangle \langle e^{\beta Q}\rangle \langle e^{\beta E_{i}}\rangle =\langle e^{-\beta \delta F}\rangle ,$$

where *β* is the inverse temperature associated with the environment, and *δF* is the standard equilibrium free energy difference. Crooks shows how, under essentially the assumption that the environment is quickly reset to a thermal state after each moment of time, the above equality indeed holds for such protocols [22].

In our experiment the system is the molecule. It is initially in a thermal state. It has, as a result of measurements, initial and final internal energies *E*_i_ and *E*_f_, respectively, and the change in internal energy is Δ*U*:=*E*_f_−*E*_i_. The time-dependent perturbation is the initial laser pulse. The energy transferred to the environment is the energy of the photon (or 0 when there is no photon). The equilibrium free energy difference is 0 in the experiment as the system Hamiltonian is the same initially and finally. All these quantities can be determined from our experimental data, as we shall describe.

Furthermore, we demonstrate how use of the quantum Jarzynski equality can be used to infer absolute energy-level shifts in molecules from the emitted photons, as opposed to just the relative shift. We thus establish that these organic molecules are a natural and powerful test bed for non-equilibrium thermodynamics and suggest how they have use in the enhanced detection of force fields.

## 2. Results

We examine the spectroscopic data from spontaneous emission of the organic molecule DBT in an anthracene crystal. The molecular system is effectively described by two energy eigenstates that are delocalized across the DBT. The molecule is coupled to a low excitation heat bath, and the joint system number state basis can be modelled as evolving unitarily under the action of

**Figure 3.**
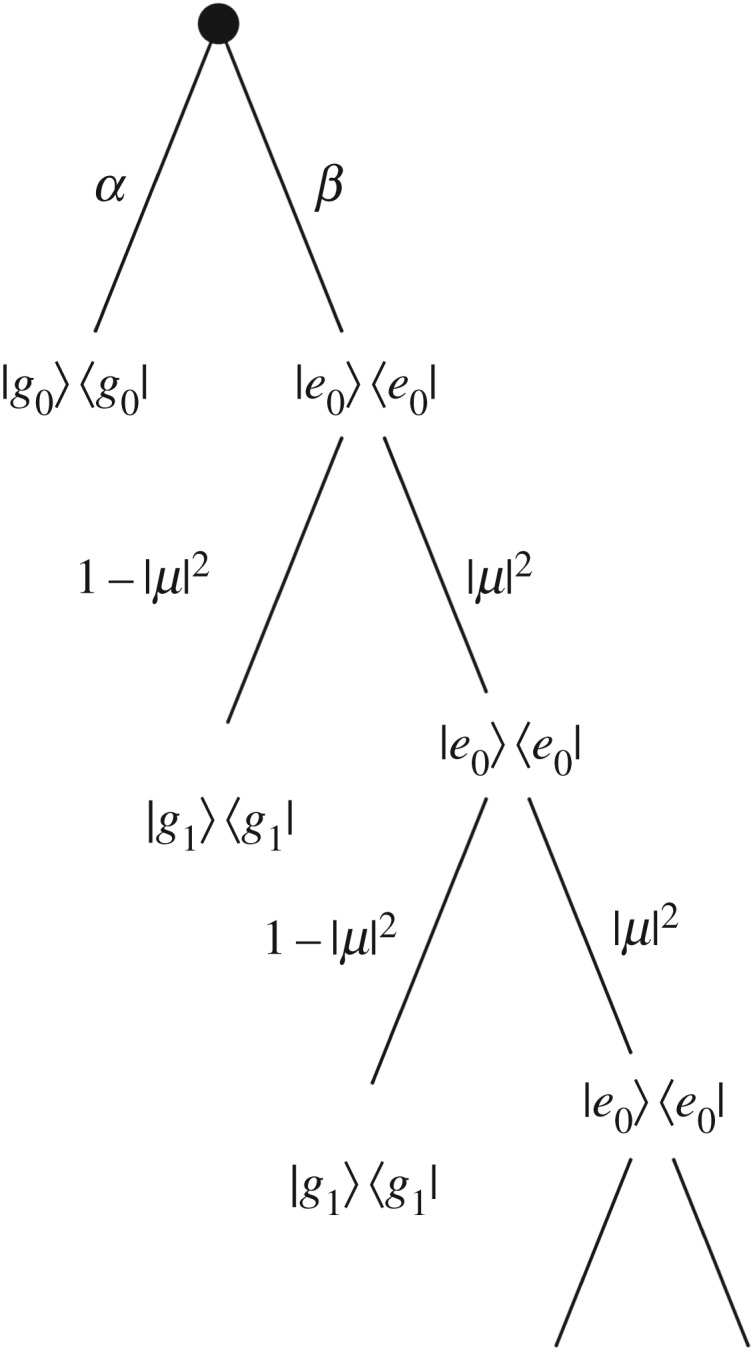
A sample of the different trajectories the system can—according to the model—take after interacting with the environment several times, after having been initialized in a mixed state. Each trajectory is formed by alternating a period of evolution under the joint Hamiltonian and then performing a projective measurement on the environment. As there is only one photon during the protocol and the probability of spontaneous excitation of the molecule is negligible, once the photon has moved into the environment there are no further branchings.

2.1 $$U=\left(\begin{array}{cccc} 1 & 0 & 0 & 0\\ 0 & \mu & -\nu^{*} & 0\\ 0 & \nu & \mu^{*} & 0\\ 0 & 0 & 0 & 1 \end{array}\right),$$

where |μ|^2^+|*ν*|^2^=1. By measuring the output environment we obtain the correct evolution of the joint system, by employing the quantum jump approach [14,28]. This coupling can be viewed as a partial swap between qubit and environment, parametrized by μ,*ν* where the overall strength of the swap is determined by |μ|^2^. It has the effect of transferring Δ*E*=*E*_2_−*E*_1_ (*E*_i_ is the energy of the *i*th level) between the molecule and the environment, with a probability dependent on the coupling strength. Within this experiment all the photons are emitted along the zero phonon line, and as such we do not observe the effects of transitions within the vibrational modes. The zero phonon line corresponds to the emission of photons from the excited state with no vibrational energy to the ground state of the molecule. With sufficient resolution of the emission spectra, it is possible to distinguish between photons due to the transition from the excited state with non-zero phonons to the ground state. If we perform periodic measurements on the output field, we can perform a partial measurement on the state of the molecule, and measure the heat flux from the molecule to the environment. One benefit of this approach is that the notion of a trajectory is built into the formulation, giving the joint system trajectories shown in figure 3. We convert the time resolved trace of the photon statistics into the heat distribution which we find to be consistent with

**Figure 4.**
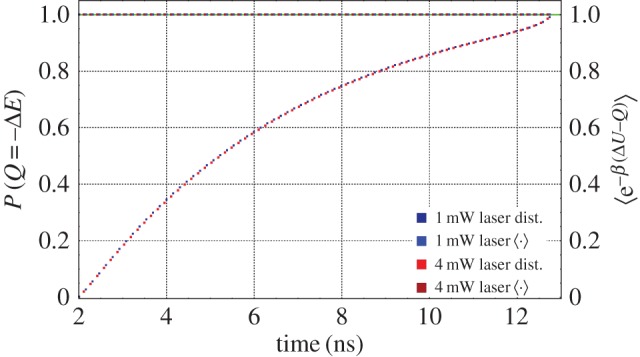
*Heat distribution and the Jarzynski equality for heat* for an organic molecule undergoing spontaneous emission at two different powers of driving laser. If we reproduce this plot for different temperatures of the sample we find qualitatively similar behaviour, with only minor numerical changes to the heat distribution. The solid green line is the theoretical prediction of 〈*e*^−*β*(Δ*U*−*Q*)^〉=1, over which we have plotted the experimentally calculated value, plotted with coloured squares. We can see that there is very good agreement between the two results, demonstrating that the Jarzynski equality for heat holds for all times during the emission. The lower dashed curve represents part of the heat distribution, which has been calculated directly from the spectroscopic data. The time evolution of the probability is interpreted using equation (2.2). The other component of the heat distribution is simply the reverse of the presented quantity, as can be seen from *P*(*Q*=−Δ*E*)=1−*P*(*Q*=Δ*E*). This is generated from the spectroscopic data by considering the cumulative number of photons emitted up to a particular time and renormalizing by the total number of photons.

2.2 $$\begin{array}{rl} P(Q=\Delta E) & =\alpha +\beta |\mu {|}^{2n}\\ \text{and}\;P(Q=-\Delta E) & =\beta (1-|\mu {|}^{2n}), \end{array}\}$$

where *α*,*β* are the initial occupation probabilities of each level and *n* indicates the number of measurements which have been performed. This can be seen by examining figure 3 and noting that the two heat values correspond to the two ‘edges’ of the tree. The heat distribution is plotted in figure 4, for two different powers of driving laser, with the sample at 4 K.

By examining the heat distribution given above, we can directly evaluate the Jarzynski equality for the heat distribution. This is directly related to the standard formulation of the Jarzynski equality through the first law of thermodynamics, Δ*U*=*W*+*Q*. In particular, we find that the system satisfies 〈*e*^−*β*(Δ*U*−*Q*)^〉=1, where Δ*U* is the change in internal energy between the initial and final states of the joint system, for all time points during the period of spontaneous emission. This demonstrates that the decay of organic molecules from an excited to the ground state is a thermodynamic process, even when it is considered at the level of single-photon emission events.

We can utilize the Jarzynski equality to detect shifts in the energy spectra of the molecule. If we consider taking the energy levels from

2.3 $$\begin{array}{rl} E_{1} & \to E_{1}+\delta \\ \text{and}\;E_{2} & \to E_{2}+\delta +\epsilon , \end{array}\}$$

where *δ* is a common shift of the energies and *ϵ* is the difference in shifts between energy levels 1 and 2. This operation produces a change in the equilibrium-free energy ($F=-k_{B}T\ln Z$) of the molecule:

2.4 $$\begin{array}{rl} \Delta F & =k_{B}T\ln\left(\frac{Z_{f}}{Z_{i}}\right)\\ & =-\delta +k_{B}T\ln\left(\frac{1+e^{-\beta \Delta E^{\prime }}}{1+e^{-\beta \Delta E}}\right). \end{array}$$

Here we have defined Δ*E*=*E*_2_−*E*_1_ and Δ*E*^′^=Δ*E*+*ϵ*. By observing the fluorescence emitted by the excited molecules, we can determine the value of both of these quantities. Combining this relation with the Jarzynski equality for heat we can determine the value of *δ*. It is possible to consider modifying the experiment discussed within this paper to investigate the feasibility of employing this theory in measuring these absolute shifts. An example alteration would be applying a magnetic field across the crystal housing the molecules to shift the energy levels. Employing equation (2.4) would allow for probing the strengths of local field effects with high sensitivity in such a scenario.

## 3. Discussion

Recently, there has been substantial interest in developing systems which can be used to test quantum thermodynamics [23,24]. Here we have demonstrated that the photon emission from organic molecules satisfies one of the fundamental relationships of the field. This is true for the single-photon emission from the molecules, at all points during the relaxation time. We believe that this provides an important platform for the study of quantum thermodynamics in real systems. Organic spectroscopy is a well-developed and established field of physics, and here we demonstrate that it is possible to tap into these resources to investigate fundamental questions in quantum thermodynamics.

In this initial formulation of the investigation, we focused on emissions along the zero phonon line. The rational behind focusing on this simplest case as an initial verification of the Jarzynski equality for heat lies in the difficulty of experimentally resolving the emissions due to transitions between the different vibro-electronic levels. Although this has been achieved in a number of experimental set-ups, the simple case discussed provides an important proof of principle. One major advantage of investigating quantum thermodynamics by utilizing fluorescence from organic molecules is that it is experimentally straightforward to relax this assumption and probe how the situation changes when we incorporate the phononic transitions. This would have the effect of creating an extra heat bath for energy transferred into the molecule to dissipate into, and would also provide for a much larger number of potential trajectories to be taken. With single-molecule spectroscopy it is possible to have sufficient resolution of the photons emitted to determine how much energy is being ‘lost’ to the phonon modes. We would then be able to determine if the Jarzynski equality for heat is still satisfied in this open system.

In this analysis, we have focused on testing the quantum Jarzynski equality for heat instead of alternative fluctuation relations such as the Crooks’ equality [16]. Although it would be of interest to test these inequalities, the nature of spontaneous emission makes them experimentally unsuitable for such investigations. This is due to the reliance of Crooks’ equality on the inverse trajectory of the process obtain the relationship. In the case of spontaneous emission this corresponds to the process of spontaneous excitation of a molecule via a photon in the environment. In order to observe this process one would need to detect single-photon fluctuations in the environment non-destructively.

In this analysis of the molecule-heat bath system, we have modelled it as initially being in a mixed state. We believe this initial condition to be reasonable as the lifetime of any entanglement between the molecule and environment is very short lived. If instead the molecule were to be left in a superposition of energy eigenstates (e.g. |*ψ*〉=|*g*0〉+|*e*0〉) after the work is done on it then in the current set-up there is no difference in the measured heat statistics relative to the corresponding de-phased mixed state. The reason this occurs is due to the repeated suppression of the coherence in the heat bath via measurement in the photon number basis and discarding the measurement outcomes. However, as the initial state before the spontaneous emission is very close to a pure product state between the molecule and the heat bath, and the Hamiltonian time evolution is unitary, the total state before measurements is approximately pure, and there will exist a basis in which measurements would not destroy coherence. As is well known [29], coherence implies extra extractable work so it would be valuable to identify how to implement this alternative basis measurement experimentally.

## 4. Material and methods

Consider the two-level molecule. We assume it is initially in a thermal state at the same temperature as the environment. In this set-up and for the modes of concern this means both the molecule and the environment are essentially in the ground state with a very small probability of a thermal excitation. The system is then pulsed by a laser which causes a swap in the occupation probabilities of the joint state. (The laser is non-resonant but there is then near-instantaneous vibrational de-excitation to the excited level of interest, figure 2.) Now the joint system undergoes evolution under its own Hamiltonian which has the effect of inducing a partial swap, parametrized by |μ|^2^, see equation (2.1). This unitary arises naturally from the Jaynes–Cummings Hamiltonian, which describes the coupling between the environment and the molecule [30]. This corresponds to the emission of a photon to the environment as the molecule is de-excited.

Framing this in the language of quantum jumps, such that we can evaluate the Jarzynski equality for heat à la [21], produces the trajectories in table 1. These trajectories are defined by considering an infinitesimal time step during which one of two events happens—either the system evolves under the joint Hamiltonian or we perform a projective measurement on the environment. The sequence of pure states produces a trajectory.

**Table 1.** *Possible trajectories* of the local state of the molecule, where *P*(|*i*〉) is the probability of initially being in state |*i*〉 and *P*_*ij*_ is the transition probability of going from state |*i*〉 to |*j*〉.

	Traj	*W*	*Q*	Δ*U*	Prob
1	|g⟩→|e⟩→|g⟩	Δ*E*	−Δ*E*	0	*P*(|*g*〉)*P*_*eg*_
2	|g⟩→|e⟩→|e⟩	Δ*E*	0	Δ*E*	*P*(|*g*〉)*P*_*ee*_
3	|e⟩→|g⟩→|e⟩	−Δ*E*	0	−Δ*E*	*P*(|*e*〉)*P*_*ge*_
4	|e⟩→|g⟩→|g⟩	−Δ*E*	Δ*E*	0	*P*(|*e*〉)*P*_*gg*_

In this framework, we consider the system to always be occupying a definite state and determine what possible evolutions each state can take. Each of these evolutions constitutes a possible trajectory the system can undertake, which have associated values of internal energy change, work and heat.

Having identified what the possible trajectories are, we must now determine which correspond to the experimental data we obtain. As we are detecting photon emission from the molecules using an avalanche photodiode (APD), in principle, we detect trajectories 1, 3 and 4. However, due to the electronics of the APD there is a delay time in receiving the signal from the photons [31]. Trajectories 3 and 4 occur on a very fast time scale due to the stimulating effect of the laser causing the transition and so are unobserved. As such, we only observe clicks in the detector due to traj. 1. The probabilities of the remaining trajectories turn out, as described below, to be determined uniquely by the initial state being assumed to be thermal together with demanding that the probabilities of all the trajectories sum to 1.

The time resolution is very high and the probability of receiving more than one photon in a given time-bin is negligible here, even taking into account the number of molecules scattered in the illuminated part of the crystal. Thus, one click means one photon.

**Figure 5.**
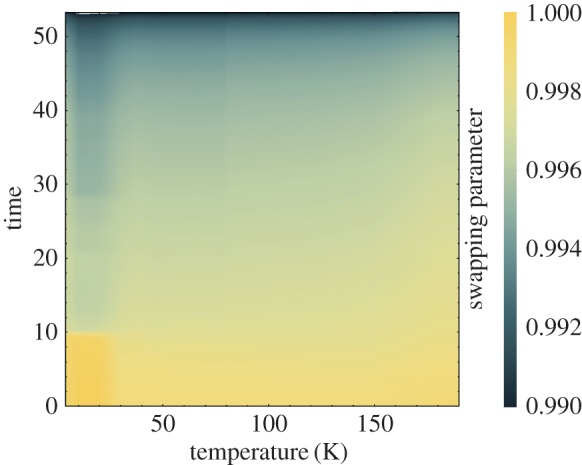
*The swapping parameter* that controls the interaction with environment is plotted as a function of temperature and the time. It can be seen that the swapping parameter is very stable for the majority of time during the experiment, indicating that the model we employ to interpret the data is sound, see equation (2.1). We utilize this in connecting the experimental data to the quantum jumps model that enables us to evaluate the Jarzynski equality for heat.

Having established the concurrence between clicks and photons, we now treat the environment as a low excitation heat bath. The emitted photons are then simply the heat flux into the bath, and by relating the cumulative probability of emission to the heat distribution, equation (2.2), we can determine the values of *α*,*β* and |μ|^2^. This information corresponds to trajectories 1 and 2, and by employing the fact that *P*(Traj 3)+*P*(Traj 4)=*P*(|*e*〉) we can explicitly calculate the Jarzynski equality for heat. By considering the distribution at *t*=0 and at long times we determine that *α*=*P*(|*g*〉) and *β*=*P*(|*e*〉). Furthermore by fitting the projected distribution to the cumulative heat distribution we determine the value of |μ|^2^, plot in figure 5. The average value of this is 〈|μ|^2^〉≈0.995, with some dependence on the temperature the experiment is carried out at. This equality is plotted in figure 4.

## 5. Conclusion

We conclude that this fluorescence experiment amounts to a scenario where Jarzynski’s equality for dissipative dynamics holds. The ‘quantumness’ of the experiment has been limited to the discreteness of the energy spectrum, as opposed to energy coherences. But the set-up lends itself naturally to also studying evolutions with energy coherences, as it deals with a qubit that can be put in a superposition by applying a laser pulse for an appropriate length of time. Moreover, the theoretical machinery which we have employed is completely valid for such superpositions. As further evidence that this experimental set-up is promising for probing quantum thermodynamics, we note/reiterate the following: (i) molecular spectroscopy is a powerful and well-established technique, (ii) the set-up maps naturally to the quantum jumps model for work and heat (used e.g. in Crook’s proof of Quantum Jarzynski [16]). A natural and exciting next step in this program is to prepare two entangled molecules to implement the quantum Maxwell’s demon of del Rio *et al.* [32]. This would build on the important first steps, we have taken with this work and reinforce the applicability of quantum thermodynamics to spectroscopy. A further area of research would be exploring the technological applications of utilizing these organic molecules to probe external fields by employing the relationship between spectroscopy and the Jarzynski equality.

## Data accessibility

All relevant data are presented in the body of the text.
